# The age of authenticity anxiety: Artificial intelligence and emerging questions for mental health

**DOI:** 10.1371/journal.pmen.0000580

**Published:** 2026-03-19

**Authors:** Adam D. Brown

**Affiliations:** Department of Psychology, New School for Social Research, Department of Psychiatry, New York University School of Medicine, New York, New York, United States of America; PLOS: Public Library of Science, UNITED KINGDOM OF GREAT BRITAIN AND NORTHERN IRELAND

Each technological era reshapes how humans experience reality, identity, and connection. Radio, television, and the internet altered the boundaries between direct and mediated experience. Generative artificial intelligence (AI), however, represents a qualitatively different shift. Unlike prior media technologies that transmitted human-created content, AI systems now generate language, images, and voices that are difficult to detect from human output. The field of mental health may therefore be entering what could be termed the age of *Authenticity Anxiety*: a phenomenon characterized by the persistent cognitive and affective preoccupation with determining whether the source of information originated from a human or a machine.

Mental health disorders already represent a leading cause of disability throughout the world, with approximately one in five adults and one in six adolescents experiencing a condition during their lifetime [1]. In this context of widespread vulnerability, the mental health implications of this rapidly shifting technological environments warrant careful attention and research. Although the mental health literature has built an important knowledge base examining the effects of social media and digital communication on well-being [2], the consequences of AI-mediated reality remain largely unexplored.

As a starting point for future research, authenticity anxiety might operate along two dimensions. The first is external: individuals increasingly assessing whether a message, image, or voice was produced by a human or generated algorithmically. The second is more internal: individuals questioning whether outputs they created with AI assistance remain authentically “theirs.” This dual process implicates core transdiagnostic factors underlying risk and resilience to mental health, including threat detection, interpersonal trust, and self-efficacy [3].

The detection of social cues, inferring intentionality, and assessing trustworthiness are highly associated with wellbeing [4]. These systems may be strained in the context of AI given the challenges one might face trying to decouple what is real and what is computer generated. A central research priority for mental health is to determine whether sustained ambiguity pertaining to sources of information increases baseline vigilance or cognitive load. Chronic hypervigilance is strongly linked with anxiety disorders, depression, and stress-related illness [4]. Does repeated uncertainty regarding authenticity produce measurable elevations in stress reactivity, attentional bias, or physiological arousal? Longitudinal and experimental designs could clarify whether exposure to AI-mediated ambiguity predicts symptom trajectories over time.

Authenticity anxiety may also amplify maladaptive cognitive processes such as rumination and self-doubt. For individuals with anxiety, depressive, or stress-related disorders, authenticity anxiety may intensify interpersonal uncertainty: Was that criticism genuine? Was that praise sincere? Did the sender intend those words? Future work would benefit from testing whether authenticity ambiguity acts as a stressor that exacerbates rumination, rejection sensitivity, or paranoid ideation in vulnerable populations. Importantly, careful differentiation is needed between adaptive skepticism in a changing digital landscape and clinically significant mistrust.

A second domain of inquiry concerns self-efficacy and agency. Self-efficacy, the belief in one’s capacity to adapt to and manage challenges, is a robust predictor of resilience and psychological functioning [5]. We are now witnessing generative AI systems produce essays, analyses, artwork, and code with profound speed. Although such tools may enhance productivity, they may also blur authorship boundaries. Does frequent reliance on AI scaffolding reduce perceived competence or creative ownership over time? Alternatively, might augmentation enhance perceived capability and reduce performance anxiety? Developmental studies will be needed to investigate impacts on adolescents and young adults, whose identities and professional trajectories are still emerging.

Social comparison processes offer another lens. Research consistently demonstrates that upward social comparison, particularly in digital environments (e.g., social media), is associated with reduced well-being [2]. AI may intensify these dynamics by obscuring the degree of machine assistance behind curated achievement. If individuals compare unaided efforts to AI-enhanced outputs presented as purely human, perceived inadequacy may increase. Empirical studies can help to shed light on whether transparency about AI assistance moderates social comparison effects and whether disclosure norms mitigate adverse mental health outcomes.

Perhaps the most consequential implications concern attachment and trust. Secure attachment relies on consistent, reliable, and authentically responsive interpersonal exchanges [6]. Trust presumes that there is a person on the other end of communication. As AI-generated text, voice, and video become increasingly prevalent, the possibility of the communication emerging from non-human origins may elicit anxiety related to trust and certainty. Key questions for future research may include: Does perceived ambiguity about authorship diminish interpersonal trust or relationship satisfaction? Does it alter attachment security? Neurobiological studies could examine whether authenticity uncertainty influences activation in social cognition and threat-detection networks and how might this vary among individuals at risk or currently diagnosed with a mental health disorder?

These concerns should not be interpreted as resistance to innovation. AI holds substantial promise for mental health, including scalable assessments, personalized medicine, and the capacity to compute large scale complex datasets [7]. The task is not to impede technological advances in AI and mental health, but to offer an emerging concept that is deserving of careful investigation.

Such findings will likely aid in the adaption and development of new therapeutics to address authenticity anxiety in the clinical context. Protective factors, such as in person contexts in which authorship is unambiguous, may reduce cognitive strain associated with authenticity uncertainty. Future clinical trials could study whether the deliberate cultivation of in person interactions reduces stress and improves well-being.

Mental health researchers long adapted to societal transformation, and the myriad of ways in which technology has shaped our lives and mental health. Generative AI introduces a new axis of uncertainty as we are now continuously confronted with information whose authorships is ambiguous. Whether authenticity anxiety becomes a transient adaptation or a sustained contributor to psychopathology, is yet to be determined, however given the pace at which AI is becoming intertwined into every facet of life, understanding this phenomenon will help those studying mental health to contribute to shaping best practices and policy within AI and mental health.
